# Late antenatal care initiation and neonatal outcomes in an ethnically diverse maternal cohort

**DOI:** 10.1093/eurpub/ckac130.243

**Published:** 2022-10-25

**Authors:** S Puthussery, Pc Tseng, E Sharma, A Harden, L Leah

**Affiliations:** 1 Maternal and Child Health Research Centre, University of Bedfordshire, Luton, UK; 2 School of Health Sciences, City University of London, London, UK; 3 Great Ormond Street Institute of Child Health, University College London, London, UK

## Abstract

**Background:**

Ethnic minority status and maternal socio-economic deprivation are linked to delayed access to health care during pregnancy. The link between late antenatal care initiation and neonatal outcomes in settings with high ethnic diversity and social disadvantage is seldom explored. This study examined associations between late antenatal care initiation (first antenatal appointment >12 weeks gestation) and neonatal outcomes of preterm birth (<37 weeks gestation) and low birth weight (<2500 g) in an ethnically diverse socially disadvantaged maternal cohort.

**Methods:**

A retrospective cross sectional study using routinely collected anonymous data of singleton births between April 2007 - March 2016 from a large UK National Health Service maternity unit in an ethnically diverse, socially disadvantaged area. Univariate and multivariate logistic regression models were used to examine the associations between late antenatal care initiation and prevalence of preterm birth and low birth weight.

**Results:**

Of the 46,307 singleton births recorded, more than one third (34.8%) were to mothers from Black African, Black Caribbean, Indian, Pakistani, and Bangladeshi mothers. Gestational week at first antenatal appointment was available for 99.31% births among which 79.2% had their first appointment at ≤ 12 weeks, 12% at 13-20 weeks, and 8.8% at > 20 weeks. Mothers who booked at 13+ weeks were significantly more likely to have a preterm and/or low birth weight baby. Compared to mothers who booked at ≤ 12 weeks, those booking at > 20 weeks were 4.08 times (95% CI: 3.29,5.07) as likely to have an extremely preterm baby (<28 weeks of gestation) and 3.12 (CI 2.66, 3.67) times as likely to have a baby born with extremely low birthweight (<1500g).

**Conclusions:**

Mothers in ethnically diverse socially deprived areas who started antenatal care late were at increased risk of adverse neonatal outcomes. Targeted intervention programmes and services are needed to support these mothers.

**Key messages:**

- Mothers who start antenatal care late are more likely to have a preterm and/or low birth weight baby in ethnically diverse socially disadvantaged areas.
- There is a need for targeted programmes and services to support mothers in ethnically diverse socially disadvantaged areas to start antenatal care on time.

